# The Proposition for Bipolar Depression Forecasting Based on Wearable Data Collection

**DOI:** 10.3389/fphys.2021.777137

**Published:** 2022-01-25

**Authors:** Pavel Llamocca, Victoria López, Milena Čukić

**Affiliations:** ^1^Computer Architecture Department, Complutense University of Madrid, Madrid, Spain; ^2^Quantitative Methods Department, Cunef University, Madrid, Spain; ^3^Institute for Technology of Knowledge, Complutense University of Madrid, Madrid, Spain; ^4^3EGA, Amsterdam, Netherlands; ^5^Department for General Physiology and Biophysics, Belgrade University, Belgrade, Serbia

**Keywords:** bipolar depression, detection, forecasting, wearables, telehealth, physiological complexity

## Abstract

Bipolar depression is treated wrongly as unipolar depression, on average, for 8 years. It is shown that this mismedication affects the occurrence of a manic episode and aggravates the overall condition of patients with bipolar depression. Significant effort was invested in early detection of depression and forecasting of responses to certain therapeutic approaches using a combination of features extracted from standard and online testing, wearables monitoring, and machine learning. In the case of unipolar depression, this approach yielded evidence that this data-based computational psychiatry approach would be helpful in clinical practice. Following a similar pipeline, we examined the usefulness of this approach to foresee a manic episode in bipolar depression, so that clinicians and family of the patient can help patient navigate through the time of crisis. Our projects combined the results from self-reported daily questionnaires, the data obtained from smart watches, and the data from regular reports from standard psychiatric interviews to feed various machine learning models to predict a crisis in bipolar depression. Contrary to satisfactory predictions in unipolar depression, we found that bipolar depression, having more complex dynamics, requires personalized approach. A previous work on physiological complexity (complex variability) suggests that an inclusion of electrophysiological data, properly quantified, might lead to better solutions, as shown in other projects of our group concerning unipolar depression. Here, we make a comparison of previously performed research in a methodological sense, revisiting and additionally interpreting our own results showing that the methodological approach to mania forecasting may be modified to provide an accurate prediction in bipolar depression.

## Introduction

Those who suffer from bipolar depressive disorder (BDD) are often misdiagnosed with unipolar depression and treated as such in average for 8 years (Singh and Rajput, 2006; Lloyd et al., 2011). In addition, there are findings suggesting that antidepressant medication can aggravate their condition (Patel et al., 2015; Robillard et al., 2021). Bipolar disorder in its various forms affects 2.4% of the population of the world (World Mental Health Survey, 2011; World Health Organization [WHO], 2017). It is a recurrent mood disorder that produces everything from extreme euphoria to severe depression. It is accompanied by alterations in thought and behavior and can produce psychotic symptoms, such as delusions and hallucinations. People who suffer from it have a high risk of suicide, 20 times more than general population (Baldessarini et al., 2020). Even with treatment, more than a third of patients will suffer at least one relapse in the first year after diagnosis and more than 60% will have a new crisis in the first 2 years. It is a disease that typically appears during adolescence or early adulthood, affecting the person throughout his/her entire life (World Health Organization [WHO], 2018). Pharmacological treatment is the main pillar in the approach to this debilitating disease. It aims to shorten crises and prevent their occurrence but the medication has serious side effects, especially at high doses. It is therefore particularly important to detect the onset of a crisis as soon as possible. Rapid treatment of a new crisis can make a big difference in the overall effectiveness. However, this early detection is very difficult from a current standardized clinical approach. At the beginning of a crisis, the symptoms and changes can be very subtle, almost impossible to notice. It is very challenging to differentiate between unipolar and bipolar depression. We showed that the detection of unipolar depression is possible by combination of machine learning and non-linear characterization of electroencephalographic (EEG) signals (Čukić et al., 2020a,b,c; Čukić and Lopez, 2020). Additionally, we demonstrated that with the same methodological approach, it is possible to differentiate between two phases of the disease, episode, and remission (Čukić et al., 2019), which can have immense significance for clinical decisions.

A common denominator at the onset of crises is the change in sleep and activity pattern. Weeks before the crisis, there are always changes in these variables (Llamocca et al., 2021). The early detection of these changes would allow for the improved possibility of social and occupational integration of the patients and would also allow for the decrease of the dose of drug needed for stabilization.

In our previous work, we dealt with prediction of the occurrence of crisis in BDD based on actigraphy measurements combined with standard reports from psychiatrists and self-report data obtained from outpatients *via* a mobile application (Llamocca et al., 2018, 2019, 2021). We used a number of methods for feature selection and a number of machine learning models that were previously applied in similar detection tasks (Llamocca et al., 2021). In conclusion, we stated that this methodology led to a real precision medicine application. It was shown that non-linear analysis of electrophysiological data could be used for monitoring state of patients with bipolar depression (Pincus, 2006; Migliorini et al., 2012; Moon et al., 2013; Nardelli et al., 2017; Byun et al., 2019). Spectral and non-linear biomarkers extracted from ECG are corresponding to the aberrations of the autonomous nervous system (ANS) of patients, but also to the severity of the disease. The relation between variability of heart rate (VHR) and depression is well described (Kemp et al., 2010, 2011, 2012). Based on the non-linear analysis of ECG (as a robust marker of vagal control), it is possible to differentiate between comorbid disorders (Kemp et al., 2012) or subtypes of depression (Kemp et al., 2014), and to point to the unreported suicidal ideation (Khandoker et al., 2017), an information of enormous significance for accurate diagnosis and effective treatment. We argue here that electrophysiological data (ECG measured by portable monitoring device) as a source of detection and forecast, properly characterized by non-linear measures, can be a game changer. We revisited and additionally interpret some of our already published data, important for developing an accurate warning system for the proximity of the crisis, allowing timely and appropriate action.

## Comparative Analysis and Discussion

**FIGURE 1:**
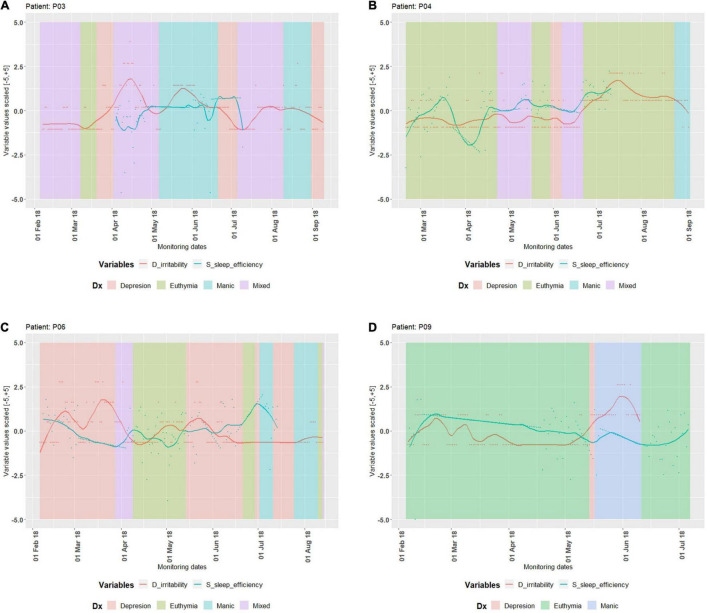
Clinical record of episodes and evolution of irritability and sleep efficiency variables in 4 patients. Patient P03 **(A)** went through all possible states and a short period in euthymic state (high-risk patient). Patient P04 **(B)** went through all possible states, however, stayed in euthymic state most of the time. Patient P06 **(C)** went through all possible states and usually got depressed with few manic episodes. Patient P09 **(D)** got depressed, manic but stayed in euthymic state most of the time. All the data depicted on the above graphs are interpolated. For detailed definitions of states, please consult original publication (Llamocca et al., 2021). On abscissa are months of collection of the data, and on ordinate are the variable values.

Our main aim in the most recent publication was to isolate relevant variables for BDD (irritability and duration of sleep turned out to be the most significant) and discover the relations between them (Llamocca et al., 2021). Being successful in the detection of unipolar depression states/phases, we applied the same method to BDD and revealed quite different dynamics of the disease with more phases than in unipolar depression (Llamocca et al., 2021). According to our results (based on accumulated clinical observations and advanced analytics), there are five distinct states with as many intermediary (bidirectional) states in BDD dynamics, described by directed graph approach (for more details of our methodology, please consult the original publication, Llamocca et al., 2021). We could not discuss all aspects of our results, due to the scope and the limitations of the journal. In this retrospective analysis together with additional interpretation of those results, we are discussing suggestions for improvement of the future methodology that might lead to a simpler solution, more attractive to clinicians. Due to very complex dynamics of bipolar depression, the personal analysis of every single case is still required, as in the classical personalized approach (Llamocca et al., 2021). Other research aiming at forecasting for BDD, also concluded that the time series extracted from similarly collected data are not possible to generalize since they are very *heterogenous*; this is actually preventing the automated mood forecasting in BDD (Moore et al., 2012). Moore and colleagues reported that for some patients the mania scores were always zero during the monitoring period, which is probably the effect of medication. Figure 1 shows the periods for defined states (depression, euthymia, manic, or mixed) in which some patients were, as well as the evolution of self-report variable D irritability and the actigraph variable S sleep efficiency. For detailed definitions of states, please consult original publication (Llamocca et al., 2021). From Figure 1, we can see different dynamics in four different patients; P03 exhibited mania and mixed state, P04 experienced euthymia and mixed state, P06 exhibited all possible states in the same period, while P09 was in the phases of long euthymia and mania, with a brief phase of depression. Although these four persons are all diagnosed with *the same clinical entity*, it is difficult to compare their dynamics as they are so different. Knowing that the mood (or states, as we labeled them) is the outcome of many complex physiological processes (that generate series of sequential data), the problem of forecasting seems to be more complicated than previously thought [in various artificial intelligence (AI) applications]. Addition of physiological complexity (fractal and non-linear) analysis to this methodology, based on our interpretation coming from Information theory, may improve the characterization of their states leading to better crisis prediction.

One of the first authors to write about the quantitative assessment strategies in mood disorders, Steven M. Pincus, introduced a novel understanding of physiological complexity, based on his rich experience with deciphering hormonal dynamics. Pincus argues that we should pay closer attention to time series that reflect essential physiological information, for there is very important history of the data, i.e., the order of samples in the time series. Pincus is the author of the Approximate Entropy algorithm (ApEn), which is a model-independent quantification of the regularity (complexity) of the data (Pincus, 1991, 1995; Pincus and Huang, 1992; Pincus and Viscarello, 1992). The fundamental difference between regularity statistics (such as, ApEn) and conventional variability measures is that the conventional approach is focusing on tasks of quantifying the degree of spread about the central value, while the order of the input data is irrelevant; whereas in irregularity statistics, ApEn tracks changes from random to very regular and the order of samples is *essential* to the algorithm (Pincus, 2003). If we shuffle the data, the intrinsic dynamics is lost, since time series reflect the essential physiological information (Pincus, 1994). Since the sequential order of mood data is relevant to diagnosis, we must use something beyond SD and means currently used in medicine, to adequately quantify the serial nature of those data (Pincus, 2003). Since ApEn and other similar entropy measures (Shannon entropy, sample entropy, multiscale entropy, etc.) started gathering attention, various research results confirmed that they are indispensable for detecting the slightest changes in the complex physiological systems, that cannot be discovered by conventional methods. In our most recent research, we detected that among those entropy-based measures, Shannon entropy yields the best result, overperforming any previously reported conventional heart rate variability (HRV) analysis (Čukić and Savić, 2021). That has sense since Shannon entropy reflects the amount of information generated by the signal (process), which can lead to discerning the system (and its states) that is functioning in a different way than the healthy one (Vajapeyam, 2014). ApEn can detect subclinical changes (the patterns that mostly remain undetected), unlike conventional time series analyses (Pincus et al., 1993). In addition, Pincus advises a combined approach of non-linear analysis of atypical heart rate (HR) dynamics and/or EEG, given the hereditary nature of bipolar disorder (Pincus, 2003, 2006). ApEn showed to be capable of detecting changes not in peaks or amplitudes, but in underlying episodic behavior, corresponding to subsystem anatomy, feedback, or coupling (Pincus and Keefe, 1992; Pincus, 1994). It can be therefore useful to predict subsequent clinical changes, such as in mood disorders. Cook used this kind of quantification (irregularity statistics) (Cook et al., 2002) to show that patients with bipolar depression exhibit changes in EEG as a reaction to antidepressant therapy. Glenn and her colleagues managed to distinguish an episode of mania or depression, in 49 patients with bipolar disorder, from the 60 days of prior euthymia, 60 days prior the change by using ApEn algorithm on time series of self-reported state (Glenn et al., 2006). Their research showed that the larger ApEn value suggests that the 60 days prior to manic episode are more disordered (*irregular*) than the 60 days prior to a depressive episode. They argued that non-linear and linear techniques of analysis may measure different underlying components of mood changes capturing patterns that are embedded in the order of the data. Their research suggested that non-linear techniques should complement traditional measures to better delineate the onset (and extent) of an episode, preventing the costly hospitalization, but also the recovery from the crisis. Moore et al. (2012) noted that the quality that seems to vary among the patients with BDD they observed is so-called *roughness*, which they addressed by application of Detrended Fluctuation Analysis (DFA), a fractal methodology belonging to the family of non-linear methods of analysis. Another important study by Migliorini et al. (2012), used portable ECG sensor embedded in T-shirts, so the patients could sleep without restraints while the constant monitoring of heart dynamics was performed. The rationale here is that underlying aberrated dynamics of ANS or cortico-vagal control (known to be disrupted in mood disorders (Rothenberg, 2007), could be used for the detection and forecasting. Again, non-linear measures showed to be superior to conventional ones (see also Gottschalk et al., 1995), and the ratings extracted from signals recorded during the whole 4 nights were more accurate than the result of the standard diagnostic procedure performed before sleep (they used ML models to differentiate between BDD and healthy controls). Faurholt-Jepsen et al. (2014) showed that self-reported (labeled “subjective”) assessment was more efficient in identifying BDD states, as in using mobile technology (smartphones) or online platforms, such as Mechanical Turk (Gillan and Whelan, 2017). Hence, some electrophysiological recording could significantly improve the chances of BDD mania prediction. Irregularity data would probably act as much more reliable features accurately representing underlying physiological information leading to better predictions. It is also important to distinguish between *detection* and *forecasting* since the latter is a much more demanding task. Having in mind that the symptoms of mood disorders are the consequences of cortico-vagal control or better, the lack of it (Rothenberg, 2007; Willner et al., 2013; Van der Kolk, 2014), non-linear measures as indicators of intrinsic dynamics (provided their sensitive quantification power) of the system are the optimal choice. Kim et al. (2013) showed that in bipolar depression, based on network analysis of EEG, there is an underlying disruption of functional connectivity.

Here, we propose two classes of methodology improvements that can result in more feasible solution for forecasting of manic episodes.

The first one is to add to the method the recording of ECG from the patients with BDD, with portable monitoring devices with medical-grade quality of signal. There are plenty of solutions, such as recording from the fingers, or from the wrists; to perform sufficiently accurate analysis, the recording from the chest is required. The signal should be analyzed by some of the abovementioned non-linear methods, irregularity statistics (entropy-based) and some form of fractal analysis. This kind of characterization of signal would eventually lead to much better prediction. The aim is to connect the values of certain measures/variables to certain diagnostic entities and their phases.

We are proposing recording of portable ECG, and not EEG (that was used for many EEG based depression detection in literature, among others, Alimardani and Boostani, 2018), aware of the problems in acquisition of the signal that can jeopardize the whole project. Telemedicine [with internet of things (IoT)] is gradually entering homes; outpatients are already using mobile applications, and the collection of data is easier than before. It is already shown that non-linear measures of ECG make it possible to differentiate between comorbid disorders (as shown in refs. Kemp et al., 2010, 2011), to delineate melancholic from non-melancholic depression (Kemp et al., 2014), or to detect the suicide ideation (Khandoker et al., 2017), which is particularly important in BDD where the risk of suicide is high (20-fold risk in comparison with controls, Baldessarini et al., 2020). Those are all immensely important for the clinician to make effective treatment decisions. In addition, since sleep is disrupted in BDD, it would make sense to measure ECG during sleep (Migliorini et al., 2012). Pincus (2003) predicted that some form of sleep recording of ECG would be the most sufficient for this task (see also Saad et al., 2019).

**FIGURE 2:**
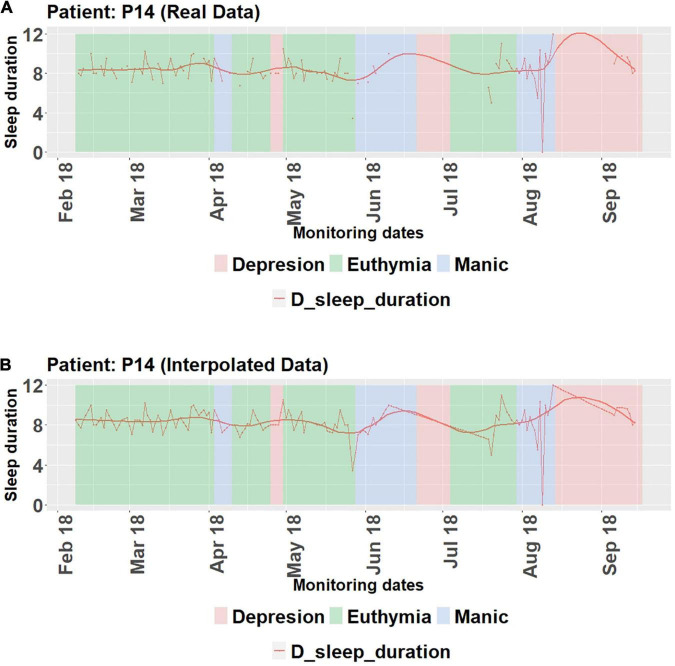
Evolution of sleep duration variable and the states the patient P14 went through. **(A)** Real data. **(B)** Interpolated data. For detailed definitions of states, please consult the original publication (Llamocca et al., 2021). On abscissa are months of collection of the data, and on ordinate are the variable values.

An example from our publication (Llamocca et al., 2021) is illustrating how variables connected to sleep (sleep duration as the most significant one) are changing in relation to the state defined for that day (either as self-reported or pronounced by a clinician, since both are included in dataset). Figure 2 shows how real data from patient P14 differ in respect to the interpolated data. We can conclude that P14 usually sleeps about 8 h in euthymic state, but this time-period varies when P14 is about to enter crisis or is already suffering from one. Figure 2 shows the periods for states in which patient P14 was, as well as the evolution of self-report variable D sleep duration (duration of their sleep).

The second part of our proposition for further improvement of approach to prediction would be in connection to ML models. We were using various forms of supervised learning to learn from the data. The authors who are dealing with more theoretical approach to computational psychiatry (Whelan and Garavan, 2014) are advocating avoidance of ‘unwarranted optimism’ by collecting more data and lowering the number of variables per person (Kohavi, 1995; Tibshirani, 1996; Ng, 1997). Relying on Bayesian approaches is recommendable. Special care should be given to dimensionality problem, which our group addressed entirely (Llamocca et al., 2021). In addition, support vector machines (SVM) might be one of the most popular models, but other methods could be used, such as embedded regularization (Whelan et al., 2013). Knowing the heterogeneity problem, we suggest introducing some of unsupervised learning methods, such as subgroup discovery (which is a binary classifier and works on labeled data) and association rule discovery (which is unsupervised ML Model), or predictive and descriptive clustering (distance-based models) (Flach, 2012). The problem with clusters can be 2-fold: either you have a trivial solution (which corresponds to overfitting in linear models, let us say clustering overfitting) that can be resolved if we penalize the large K, or if we fix the number of clusters K in advance; the problem cannot be solved for large datasets (but a typical dataset is not large). Soft clustering generalizes the notion of partition, in the same way that a probability estimator generalizes a classifier (Flach, 2012). With the abovementioned suggestion, the algorithm can learn from the (properly characterized) data. We can conclude what the subgroups are and what the relations between present instances are, so we can try to interpret them in the light of information theory approach to physiological processes.

Busk et al. (2020) used a similar manner of collecting the data, with different items in the questionnaire. They tested the feasibility of forecasting daily subjective mood scores based on daily self-assessment from 84 patients with bipolar disorders via smartphone in a randomized clinical trial. Combined historic data and currently collected data improved forecasting and used Hierarchical Bayesian approach, a multi-task learning method. They used data from different subjects as additional cases to learn. Ordinal regression (or ordinal classification) is a method of predicting a discrete variable that has a relative ordering of the possible outcomes. First, they started with 1-day forecast with several scenarios (two time-series cross-validation experiments) and applied best model to evaluate 7-day forecast. When increasing the forecast horizon, forecast errors also increased and the forecast regression shifted toward the mean of data distribution; the best model used a 4-day history of self-assessment. Interestingly, authors used similar organization of the dataset that is usually used for entropy-based analysis of physiological data, discussed above; maybe the historicity of the data would be the key for successful forecasting. Besides, some shift in ML models used for much needed realistic forecasting includes much preferred unsupervised learning or functional data analysis (Wang et al., 2016). Table 1 is offering some recommended techniques with our justification.

**TABLE 1:** Methods of detection and prediction of bipolar depression, used in the literature and recommended, with practical explanations and citation.

	Methods used	Methods recommended	Practical explanation
**Detection**			
1	Patient’s medical history, scales, epidemiological data	Electrophysiological signals (EEG, ECG.)	Coley et al. (2021) showed that epidemiological data cannot help in prediction; physiological dynamics can
2	EEG based detection of depression	ECG based detection of depression	Portable monitoring devices for EEG are still few and expensive, those for ECG are more accessible
3	Sub-bands analysis	Broad-band analysis	There is no physiological explanation of support for importance of sub-bands
4	Small sample sizes	Larger (collaborative) sample sizes	Existing effect can be better detected with decent effect size, demonstrating practically useful results
5	Big number of variables per person	Keep the ratio under 10	Unwarranted optimism (Ng, 1997; Whelan et al., 2013)
6	ECG detected from fingers or wrist	ECG detected from the chest	Medical-grade quality of signal leads to higher accuracies of detection/prediction
7	Conventional time and frequency measures of HRV	Fractal and non-linear measures of HRV (HFD, DFA, entropy based measures, Poincare plots.)	Effect sizes for non-linear detection overperform conventional measures detection for a whole magnitude on scale (corrected Cohen’s d∼ 0.2 vs. 7.7, Čukić and Savić, 2021)
8	Aggressive pre-processing of electrophysiological signals	Using artifact free unfiltered signals, or Deep Learning of raw signal to correct for artifacts	By overly filtering and Fourier’s decomposition (reductionistic approach) important information about history of data (sequentionality important for regularity statistics) is lost
**Prediction/Forecasting**			
1	Frequentist statistics	Bayesian approach	Improved accuracy for real life use
2	Historical medical data	Non-linear measures as feature extraction	Features based on complex systems dynamics approach lead to realistic results
3	Variation around mean values	Complex variability (physiological complexity)	Irregularity statistics is much better suitable for quantifying physiological dynamics which is non-stationary, non-linear and noisy
4	SVM and other popular ML models	LASO embedded regularisation, unsupervised learning, clustering, FDA	Practically useful prediction
5	Outliers removal	Deep learning on raw data (ECG)	Keeping the intrinsic structure of the data intact
6	Feature extraction based on t (ANOVA)	PCA, GA or FDA	Much better sensitivity and specificity
7	Non-existing external validation	ROC curve application (AUC)	More realistic results

We hope that an improved research methodology, based on abovementioned comparison and analysis, would eventually lead to a much better theragnostic and improve the quality of life of patients.

## Author Contributions

VL developed the idea for research. PL, VL, and MČ performed the research, wrote the manuscript, and reviewed the manuscript. PL and VL collected and analyzed the data. PL generated figures. All authors contributed to the article and approved the submitted version.

## Conflict of Interest

The authors declare that the research was conducted in the absence of any commercial or financial relationships that could be construed as a potential conflict of interest.

## Publisher’s Note

All claims expressed in this article are solely those of the authors and do not necessarily represent those of their affiliated organizations, or those of the publisher, the editors and the reviewers. Any product that may be evaluated in this article, or claim that may be made by its manufacturer, is not guaranteed or endorsed by the publisher.

## Abbreviations

EEG: Electroencephalogram
ECG: Electrocardiogram
HRV: Heart rate variability
CVC: Cardio-vagal control
HFD: Higuchi Fractal Dimension
DFA: Detrended Fluctuation Analysis
ROC: Receiver operating characteristic
AUC: Area under the curve
PCA: Principal component analysis
GA: Genetic algorithm
FDA: Functional data analysis
LASO: the name of the algorithm, a type of linear regression that uses shrinkage
ANOVA: Analysis of variance
SVM: Support vector machines.

## References

[B1] Alimardani F. Boostani R. (2018). DB-FFR: a modified feature selection algorithm to improve discrimination rate between bipolar mood disorder (BMD) and schizophrenic patients. *Iran. J. Sci. Technol. Trans. Electric. Eng.* 42 251–260. 10.1007/s40998-018-0060-x

[B2] Baldessarini R. J. Vázquez G. H. Tondo L. (2020). Bipolar depression: a major unsolved challenge. *Int. J. Bipolar Disord.* 8:1. 10.1186/s40345-019-0160-1 31903509 PMC6943098

[B3] Busk J. Faurholt-Jepsen M. Frost M. Bardram J. E. Kessing L. V. Winther O. (2020). Forecasting mood in bipolar disorder from smartphone self-assessments: hierarchical bayesian approach. *JMIR Mhealth Uhealth* 8:e15028. 10.2196/15028 32234702 PMC7367518

[B4] Byun S. Kim A. Y. Jang E. H. Kim S. Choi K. W. Yu H. Y. (2019). Entropy analysis of heart rate variability and its application to recognize major depressive disorder: a pilot study. *Technol. Health Care* 27 407–424. 10.3233/THC-199037 31045557 PMC6597986

[B5] Coley R. Y. Boggs J. M. Beck A. Simon G. E. (2021). Predicting outcomes of psychotherapy for depression with electronic health record data. *J. Affect. Disord. Rep.* 6:100198. 10.1016/j.jadr.2021.100198 34541567 PMC8448296

[B6] Cook I. A. Leuchter A. F. Morgan M. Witte E. Stubbeman W. F. Abrams M. (2002). Early changes in prefrontal activity characterize clinical responders to antidepressants. *Neuropsychopharmacology* 27 120–131. 10.1016/S0893-133X(02)00294-4 12062912

[B7] Čukić M. Lopez V. (2020). “On mistakes we made in prior computational psychiatry data driven approach projects and how they jeopardize translation of those findings in clinical practice,” in *IntelliSys Conference, Amsterdam 3-5 September 2020. “Advances in Intelligent Systems and Computing”*, eds Arai K. Kapoor S. Bhatia R. (Berlin: Springer Verlag), 493–510. 10.1007/978-3-030-55190-2_37

[B8] Čukić M. Savić D. (2021). Another Godot who is still not coming: more on biomarkers for depression. *Revista de Psiquiatría y Salud Mental* 10.1016/j.rpsm.2021.12.006 (in press) 35840283

[B9] Čukić M. Stokić M. Radenković S. Ljubisavljević M. Simić S. Savić D. (2019). Nonlinear analysis of EEG complexity in episode and remission phase of recurrent depression. *Int. J. Methods Psychiatry Res.* 29:e1816. 10.1002/MPR.1816 31820528 PMC7301286

[B10] Čukić M. López V. Pavón J. (2020a). Classification of depression through resting-state electroencephalogram as a novel practice in psychiatry. *J. Med. Internet Res.* 22:e19548. 10.2196/19548 33141088 PMC7671839

[B11] Čukić M. Stokić M. Radenković S. Ljubisavljević M. Simić S. Savić D. (2020b). Nonlinear analysis of EEG complexity in episode and remission phase of recurrent depression. *Int. J. Methods Psychiatr. Res.* 29:e1816. 10.1002/MPR.1816 31820528 PMC7301286

[B12] Čukić M. Stokić M. Simić S. Pokrajac D. (2020c). The successful discrimination of depression from EEG could be attributed to proper feature extraction and not to a particular classification method. *Cogn. Neurodyn.* 14 443–455. 10.1007/s11571-020-09581-x 32655709 PMC7334335

[B13] Faurholt-Jepsen M. Vinberg M. Frost M. Christensen E. M. Bardram J. Kessing L. V. (2014). Daily electronic monitoring of subjective and objective measures of illness activity in bipolar disorder using smartphones–the MONARCA II trial protocol: a randomized controlled single-blind parallel-group trial. *BMC Psychiatry* 14:309. 10.1186/s12888-014-0309-5 25420431 PMC4247697

[B14] Flach P. (2012). *Machine Learning: The Art And Science Of Algorithms That Make Sense Of Data.* Cambrdige: Cambridge University Press.

[B15] Gillan C. M. Whelan R. (2017). What big data can do for treatment in psychiatry. *Curr. Opin. Behav. Sci.* 18 34–42.

[B16] Glenn T. Whybrow P. C. Rasgon N. Grof P. Alda M. Baethge C. (2006). Approximate entropy of self-reported mood prior to episodes in bipolar disorder. *Bipolar Disord.* 8 424–429. 10.1111/j.1399-5618.2006.00373.x 17042880

[B17] Gottschalk A. Bauer M. S. Whybrow P. C. (1995). Evidence of chaotic mood variation in bipolar disorder. *Arch. Gen. Psychiatry* 52 947–959. 10.1001/archpsyc.1995.03950230061009 7487343

[B18] Kemp A. (2011). Depression, antidepressant treatment and the cardiovascular system. *Acta Neuropsychiatr.* 23 82–83. 10.1111/j.1601-5215.2011.00535.x

[B19] Kemp A. H. Quintana D. S. Felmingham K. L. Matthews S. Jelinek H. F. (2012). Depression, comorbid anxiety disorders, and heart rate variability in physically healthy, unmedicated patients: implications for cardiovascular risk. *PLoS One* 7:e30777. 10.1371/journal.pone.0030777 22355326 PMC3280258

[B20] Kemp A. H. Quintana D. S. Gray M. A. Felmingham K. L. Brown K. Gatt J. M. (2010). Impact of depression and antidepressant treatment on heart rate variability: a review and meta-analysis. *Biol. Psychiatry* 67 1067–1074. 10.1016/j.biopsych.2009.12.012 20138254

[B21] Kemp A. H. Quintana D. S. Malhi G. S. (2011). Effects of serotonin reuptake inhibitors on heart rate variability: methodological issues, medical comorbidity, and clinical relevance. *Biol. Psychiatry* 69 e25–e26. 10.1016/j.biopsych.2010.10.10.0365 21353666

[B22] Kemp A. H. Quintana D. S. Quinn C. R. Hopkinson P. Harris A. W. (2014). Major depressive disorder with melancholia displays robust alterations in resting state heart rate and its variability: implications for future morbidity and mortality. *Front. Psychol.* 5:1387. 10.3389/fpsyg.2014.01387 25505893 PMC4245890

[B23] Khandoker A. H. Luthra V. Abouallaban Y. Saha S. Ahmed K. I. Mostafa R. (2017). Predicting depressed patients with suicidal ideation from ECG recordings. *Med. Biol. Eng. Comput.* 55 793–805. 10.1007/s11517-016-1557-y 27538398

[B24] Kim D. J. Bolbecker A. R. Howell J. Rass O. Sporns O. Hetrick W. P. (2013). Disturbed resting state EEG synchronization in bipolar disorder: a graph-theoretic analysis. *NeuroImage Clin.* 2 414–423. 10.1016/j.nicl.2013.03.007 24179795 PMC3777715

[B25] Kohavi R. (1995). A study of cross-validation and bootstrap for accuracy estimation and model selection. *IJCAI* 14 1137–1145.

[B26] Llamocca P. Čukić M. Junestrand A. Urgelés D. López V. L. (2018). “Data source analysis in mood disorder research,” in *In XVIII Conferencia de la Asociación Española para la Inteligencia Artificial (CAEPIA 2018) 23-26 de octubre de 2018 Granada* (España: Asociación Española para la Inteligencia Artificial (AEPIA)), 893–898.

[B27] Llamocca P. López V. Santos M. Čukić M. (2021). Personalized characterization of emotional states in patients with bipolar disorder. *Mathematics* 9:1174. 10.3390/math9111174

[B28] Llamocca P. Urgelés D. Cukic M. Lopez V. (2019). “Bip4Cast: some advances in mood disorders data analysis,” in *Proceedings of the 1st International Alan Turing Conference on Decision Support and Recommender Systems, London* (Berlin: Springer), 5–10.

[B29] Lloyd L. C. Giaroli G. Taylor D. Tracy D. K. (2011). Bipolar depression: clinically missed, pharmacologically mismanaged. *Ther. Adv. Psychopharmacol.* 1 153–162. 10.1177/2045125311420752 23983940 PMC3736904

[B30] Migliorini M. Mendez M. O. Bianchi A. M. (2012). Study of heart rate variability in bipolar disorder: linear and non-linear parameters during sleep. *Front. Neuroeng.* 4:22. 10.3389/fneng.2011.00022 22291638 PMC3254053

[B31] Moon E. Lee S. H. Kim D. H. Hwang B. (2013). Comparative study of heart rate variability in patients with schizophrenia, bipolar disorder, post-traumatic stress disorder, or major depressive disorder. *Clin. Psychopharmacol. Neurosci.* 11 137–43. 10.9758/cpn.2013.11.3.137 24465250 PMC3897762

[B32] Moore P. J. Little M. A. McSharry P. E. Geddes J. R. Goodwin G. M. (2012). Forecasting depression in bipolar disorder. *IEEE Trans. Biomed. Eng.* 59 2801–2807. 10.1109/TBME.2012.2210715 22855220

[B33] Nardelli M. Lanata A. Bertschy G. Scilingo E. P. Valenza G. (2017). Heartbeat complexity modulation in bipolar disorder during daytime and nighttime. *Sci. Rep.* 7:17920. 10.1038/s41598-017-18036-z 29263393 PMC5738374

[B34] Ng A. Y. (1997). Preventing “Overfitting” of Cross-Validation Data. Presented at the 14th International Conference on Machine Learning (ICML) (1997). Available Online at: http://robotics.stanford.edu/ãng/papers/cv-final.pdf

[B35] Patel R. Reiss P. Shetty H. Broadbent M. Stewart R. McGuire P. (2015). Do antidepressants increase the risk of mania and bipolar disorder in people with depression? A retrospective electronic case register cohort study. *BMJ Open* 5:e008341. 10.1136/bmjopen-2015-008341 26667012 PMC4679886

[B36] Pincus S. (1995). Approximate entropy (ApEn) as a complexity measure. *Chaos* 5 110–117. 10.1063/1.166092 12780163

[B37] Pincus S. M. (1991). Approximate entropy as a measure of system complexity. *Proc. Natl. Acad. Sci. U. S. A.* 88 2297–2301. 10.1073/pnas.88.6.2297 11607165 PMC51218

[B38] Pincus S. M. (1994). Greater signal regularity may indicate increased system isolation. *Math. Biosci.* 122 161–181. 10.1016/0025-5564(94)90056-6 7919665

[B39] Pincus S. M. (2003). Quantitative assessment strategies and issues for mood and other psychiatric serial study data. *Bipolar Disord.* 5 287–294. 10.1034/j.1399-5618.2003.00036.x 12895206

[B40] Pincus S. M. (2006). Approximate entropy as a measure of irregularity for psychiatric serial metrics. *Bipolar Disord.* 8 430–440. 10.1111/j.1399-5618.2006.00375.x 17042881

[B41] Pincus S. M. Cummins T. R. Haddad G. G. (1993). Heart rate control in normal and aborted-SIDS infants. *Am. J. Physiol. Regul. Integr. Comp. Physiol.* 264 R638–R646. 10.1152/ajpregu.1993.264.3.R638 8457020

[B42] Pincus S. M. Goldberger A. L. (1994). Physiological time-series analysis: what does regularity quantify? *Am. J. Physiol. Heart Circ. Physiol.* 266 H1643–H1656. 10.1152/ajpheart.1994.266.4.H1643 8184944

[B43] Pincus S. M. Huang W. M. (1992). Approximate entropy: statistical properties and applications. *Commun. Stat. Theory Methods* 21 3061–3077. 10.1080/03610929208830963

[B44] Pincus S. M. Keefe D. L. (1992). Quantification of hormone pulsatility via an approximate entropy algorithm. *Am. J. Physiol. Endocrinol. Metab.* 262 E741–E754. 10.1152/ajpendo.1992.262.5.E741 1590385

[B45] Pincus S. M. Mulligan T. Iranmanesh A. Gheorghiu S. Godschalk M. Veldhuis J. D. (1996). Older males secrete luteinizing hormone and testosterone more irregularly, and jointly more asynchronously, than younger males. *Proc. Natl. Acad. Sci. U. S. A.* 93 14100–14105. 10.1073/pnas.93.24.14100 8943067 PMC19501

[B46] Pincus S. M. Viscarello R. R. (1992). Approximate entropy: a regularity measure for fetal heart rate analysis. *Obstetr. Gynecol.* 79 249–255. 1731294

[B47] Robillard R. Saad M. Ray L. B. BuJáki B. Douglass A. Lee E. K. (2021). Selective serotonin reuptake inhibitor use is associated with worse sleep-related breathing disturbances in individuals with depressive disorders and sleep complaints: a retrospective study. *J. Clin. Sleep Med.* 17 505–513. 10.5664/jcsm.8942 33118928 PMC7927326

[B48] Rothenberg J. (2007). Cardiac vagal control in depression: a critical analysis. *Biol. Psychol.* 74 200–211. 10.1016/j.biopsycho.2005.08.010 17045728

[B49] Saad M. Ray L. B. Bujaki B. Parvaresh A. Palamarchuk I. De Koninck J. (2019). Using heart rate profiles during sleep as a biomarker of depression. *BMC Psychiatry* 19:168. 10.1186/s12888-019-2152-1 31174510 PMC6554996

[B50] Singh T. Rajput M. (2006). Misdiagnosis of bipolar disorder. *Psychiatry (Edgmont)* 3 57–63. PMC2945875 20877548

[B51] Tibshirani R. (1996). Regression shrinkage and selection via the lasso. *J. R. Stat. Soc. B (Methodol.)* 58 267–288. 10.1111/j.2517-6161.1996.tb02080.x

[B52] Vajapeyam S. (2014). Understanding Shannon’s entropy metric for information. *arXiv* [Preprint]. Available Online at: https://arxiv.org/abs/1405.2061.

[B53] Van der Kolk B. (2014). *The Body Keeps The Score: Mind, Brain And Body In The Transformation Of Trauma.* New York: Penguin.

[B54] Wang J.-L. Chiou J.-M. Müller H.-G. (2016). Review of functional data analysis. *Annu. Rev. Stat. Appl.* 3 257–295. 10.1146/annurev-statistics-041715-033624

[B55] Whelan R. Garavan H. (2014). When optimism hurts: inflated predictions in psychiatric neuroimaging. *Biol. Psychiatry* 75 746–748. 10.1016/j.biopsych.2013.05.014 23778288

[B56] Willner P. Scheel-Krüger J. Belzung C. (2013). The neurobiology of depression and antidepressant action. *Neurosci. Biobehav. Rev.* 37 2331–2371. 10.1016/j.neubiorev.2012.12.007 23261405

[B57] World Health Organization [WHO] (2017). *Depression And Other Common Mental Disorders.* Available Online at: http://apps.who.int/iris/bitstream/10665/254610/1/WHO-MSD-MER-2017.2-eng.pdf

[B58] World Health Organization [WHO] (2018). *International Suicide Rates, 2018.* Available Online at: http://www.who.int/gho/mental_health/suicide_ratescrude/en/

